# Fatal Reversible Cerebral Vasoconstriction Syndrome: A Complication of Postpartum Haemorrhagic Shock

**DOI:** 10.7759/cureus.84979

**Published:** 2025-05-28

**Authors:** Philipp Becker, Naomi S Stierle, Miriam Wick, Mailin Waldecker, Mihaela Fluri, Werner Z'Graggen, Franca Wagner

**Affiliations:** 1 Department of Diagnostic and Interventional Neuroradiology, Inselspital, University Hospital Bern, Bern, CHE; 2 Department of Emergency Medicine, Inselspital, University Hospital Bern, Bern, CHE; 3 Department of Obstetrics and Gynecology, Inselspital, University Hospital Bern, Bern, CHE; 4 Department of Neurosurgery, Inselspital, University Hospital Bern, Bern, CHE; 5 Department of Neurology, Inselspital, University Hospital Bern, Bern, CHE; 6 Department of Otorhinolaryngology, Head and Neck Surgery, Inselspital, University Hospital Bern, Bern, CHE

**Keywords:** cerebral ischaemia, intracranial vasospasm, massive blood transfusion, neurocritical care, postpartum haemorrhagic shock, reversible cerebral vasoconstriction syndrome

## Abstract

Reversible cerebral vasoconstriction syndrome (RCVS) is a rare but serious cerebrovascular disorder characterised by transient narrowing of the cerebral arteries, triggered by internal or external stressors. We present a case of RCVS in a 28-year-old female patient 10 days postpartum, after a haemorrhagic shock. The initial acute management of the abdominal haemorrhagic shock syndrome included massive transfusion of blood products along with vasopressors and crystalloids. Although her condition stabilised initially, she later exhibited severe neurological deficits of unknown cause, prompting further investigations and imaging. An immediate native computed tomography (CT) scan revealed an infarct demarcation in the posterior territory of the right middle cerebral artery (MCA) and the watershed zones bilaterally, accompanied by oedema of the right cerebral hemisphere. Intracranial CT angiography showed severe arterial narrowing, which was most pronounced in the right MCA and its branches. Digital subtraction angiography (DSA) confirmed marked intracranial vasospasm consistent with the diagnosis of a RCVS. Intra-arterial spasmolysis was performed, but the patient's neurological condition continued to deteriorate. A further CT scan showed progressive brain oedema and new infarct demarcation in the basal ganglia on the right side with persistent vasospasm. The spasmolysis was repeated with only moderate success. Magnetic resonance imaging (MRI) of the brain and spine was performed to evaluate the prognosis and exclude additional causes of the severe vasospasm. There, progressive infarct demarcation due to the persistent refractory vasospasm and signs of a secondary hypoxic encephalopathy were identified. Despite aggressive interventions, the patient’s condition deteriorated, and she passed away six days after the haemorrhagic event, highlighting the severity of RCVS in this context. This case underscores the need for prompt recognition, rapid intervention, and comprehensive management strategies for patients with severe vasospasm following postpartum haemorrhagic shock. It also highlights the impact of intracranial vasospasm resulting in RCVS as a potential critical complication of life-saving treatments such as massive transfusion and vasoactive drug administration, particularly in postpartum women.

## Introduction

Patients with reversible cerebral vasoconstriction syndrome (RCVS) typically present with the chief complaint of severe headaches (“thunderclap headache”) and frequent neurological deficits, which may be persistent or transient, including hemiparesis, aphasia, visual field defects, and seizures [1-3]. The classical finding on computed tomography (CT), magnetic resonance imaging (MRI) and digital subtraction angiography (DSA) is reversible segmental and multifocal vasoconstriction of the cerebral arteries [1,2,4]. The outcome of RCVS varies widely; some patients recover fully while others may face severe disability or even die [2,5,6].

A multitude of RCVS triggers have been identified including female sex, pregnancy, vasoactive medications, illicit drug use, blood transfusions, and various physical and mechanical causes [1,4,7,8]. Since the underlying pathophysiology of RCVS remains poorly understood, recognising and characterising these trigger factors are essential for prompt detection and improved outcomes. Consequently, the development of effective detection strategies that integrate clinical evaluation and diagnostic imaging becomes even more important.

In the acute management of haemorrhagic shock, preventing exsanguination takes priority. Massive transfusion protocols are often life-saving, despite the risk of serious complications. One such complication is RCVS, a potentially fatal neurological event. In the literature, transfusion-associated RCVS is described as “rare,” suggesting underestimation of its true incidence [5,9]. Clinical overlap with other neurological emergencies, such as subarachnoid haemorrhage, may contribute to its misdiagnosis. To our knowledge, no specific incidence data are available, likely due to its rarity and the challenges of studying uncommon transfusion-related reactions.

Clinicians treating haemorrhagic shock must also consider the risk of triggering RCVS, especially in postpartum women [3,8-11]. Obstetric emergencies often require massive transfusions, and the combination of postpartum physiological changes, severe blood loss, and haemodynamic stress creates a clinical setting in which RCVS may arise. This underscores the need for heightened vigilance and early neurological assessment in high-risk patients. Greater awareness of transfusion-associated RCVS is crucial to improve timely diagnosis and reduce its impact in vulnerable populations.

Our case report highlights the fatal outcome of a 28-year-old female patient who developed RCVS following a massive transfusion due to haemorrhagic shock 10 days postpartum. Despite interventions, the patient’s condition deteriorated rapidly, and she died six days after the initial haemorrhagic event. This underscores the importance of recognising trigger factors for RCVS, especially massive transfusions, which despite being life-saving, can result in severe cerebrovascular complications. This case is significant because, to our knowledge, it’s the first fatal instance of transfusion-related RCVS in a previously healthy postpartum woman. This contrasts with previous reports that typically involve patients with chronic severe anaemia or non-fatal outcomes. It emphasises the need for increased clinical awareness and early neurological assessment in high-risk scenarios.

## Case presentation

A 28-year-old female patient was referred to our university hospital with haemorrhagic shock 10 days after the delivery of her second child via caesarean section. The primary caesarean section had been performed electively due to a known high-risk coronary artery anomaly, specifically a malignant origin variant of the right coronary artery (RCA) with an interarterial course between the aorta and the pulmonary artery as well as kinking. The patient had previously undergone an uncomplicated vaginal delivery and had no known pre-existing medical conditions. However, following the postpartum death of her sister from myocardial infarction, a cardiological evaluation was undertaken, and - based on a shared decision - a caesarean section was planned and executed without complications under peridural anaesthesia (PDA). After discharge, the patient experienced a severe headache at home on the fifth day postpartum, which she self-managed with analgesics. 

On the 10th postpartum day, she developed recurrent vaginal bleeding accompanied by a syncopal episode. Emergency services were activated, and the patient was found to be in a critical condition with presyncope and profound hypotension (systolic blood pressure of 60 mmHg). She was transferred to a secondary care centre, where initial evaluation revealed bradycardia and dyspnoea, requiring administration of atropine. Abdominal ultrasound (US) confirmed the presence of free intraperitoneal fluid. The initial emergency management included administration of three units of packed red blood cells (RBCs), crystalloids (three litres), and oxytocin (5 IU). Given the suspicion of acute haemorrhagic abdominal shock syndrome, the patient was urgently referred to our tertiary care centre. Following her arrival, a second US confirmed a large volume of free fluid in the abdomen and a mild pericardial effusion. The patient’s condition deteriorated rapidly, necessitating immediate intubation. A re-laparotomy and abdominal packing were performed in the emergency room without prior imaging. Approximately four liters of blood, some of it already clotted, was found intra-abdominally, with blood oozing from a corner of the uterotomy. The patient was transferred to the operating room where the uterotomy was re-opened and re-sutured. 

The patient received a total of seven units of packed RBCs, four units of fresh frozen plasma, 2 g of fibrinogen, and 4 g of calcium. Repetitive viscoelastic testing was employed to guide transfusion management. To stabilise her blood pressure, norepinephrine was continuously administered via a perfusor at an average rate of 0.8-1.3 µg/kg/min during the entire period of active treatment. The metabolic work-up revealed a severe high anion gap lactic acidosis with a pH of 7.040 (normal arterial pH: 7.35-7.45), markedly low bicarbonate, and elevated lactate levels up to 17 mmol/L (normal reference range: 0.5-2.0 mmol/L), indicating tissue hypoperfusion.

Post-operatively, the patient was sedated and monitored in our intensive care unit overnight. Following the cessation of sedation the next morning, the patient’s Glasgow Coma Scale (GCS) score was seven points. The GCS is a clinical score used to assess a patient’s level of consciousness based on eye, verbal, and motor responses, with scores ranging from three (deep coma) to 15 (fully alert). A score of seven indicates a severe impairment of consciousness and is generally consistent with a comatose state. She exhibited left upward gaze deviation and intermittent seizure-like activity, raising significant concerns about an underlying neurological complication. No definitive cause for her condition could be identified at that point. Further diagnostic imaging was warranted to determine the aetiology of her symptoms.

**Figure 1:**
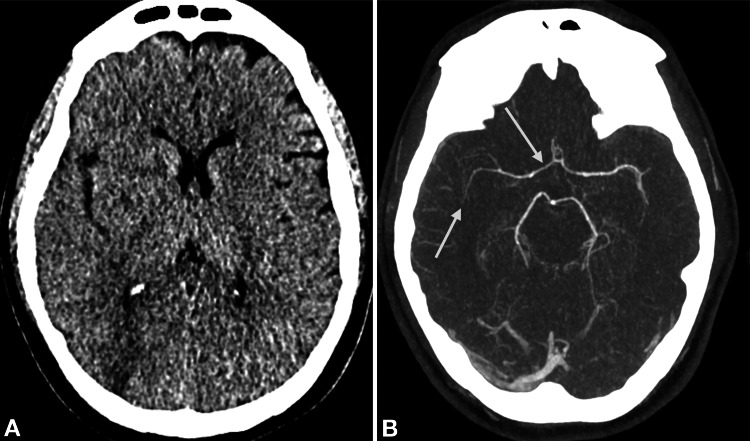
Initial native computed tomography and computed tomography angiography of the brain (A) Native computed tomography shows demarcated ischaemia in the right posterior and inner border zone, as well as frontoparietally on the right-hand side, along with an oedematous right cerebral hemisphere. (B) Computed tomography angiography reveals severe, multifocal vessel narrowing (arrows), most pronounced in the right middle cerebral artery.

CT imaging was performed to rule out the underlying pathology of persistent severe reduction in vigilance. The native CT scan of the brain with CT angiography (Figure 1A) already showed demarcated ischaemia in the right posterior and inner border zone, as well as frontoparietally on the right-hand side.

Additionally, the right cerebral hemisphere exhibited oedema and constricted sulci, accompanied by a stretched optic nerve. More pronounced changes were observed on the left side, including optic nerve-head protrusion (papillary oedema), suggesting elevated intracranial pressure. The CT angiography (Figure 1B) confirmed the severe generalized vasospasms, particularly in the medial cerebral artery on the right side.

**Figure 2:**
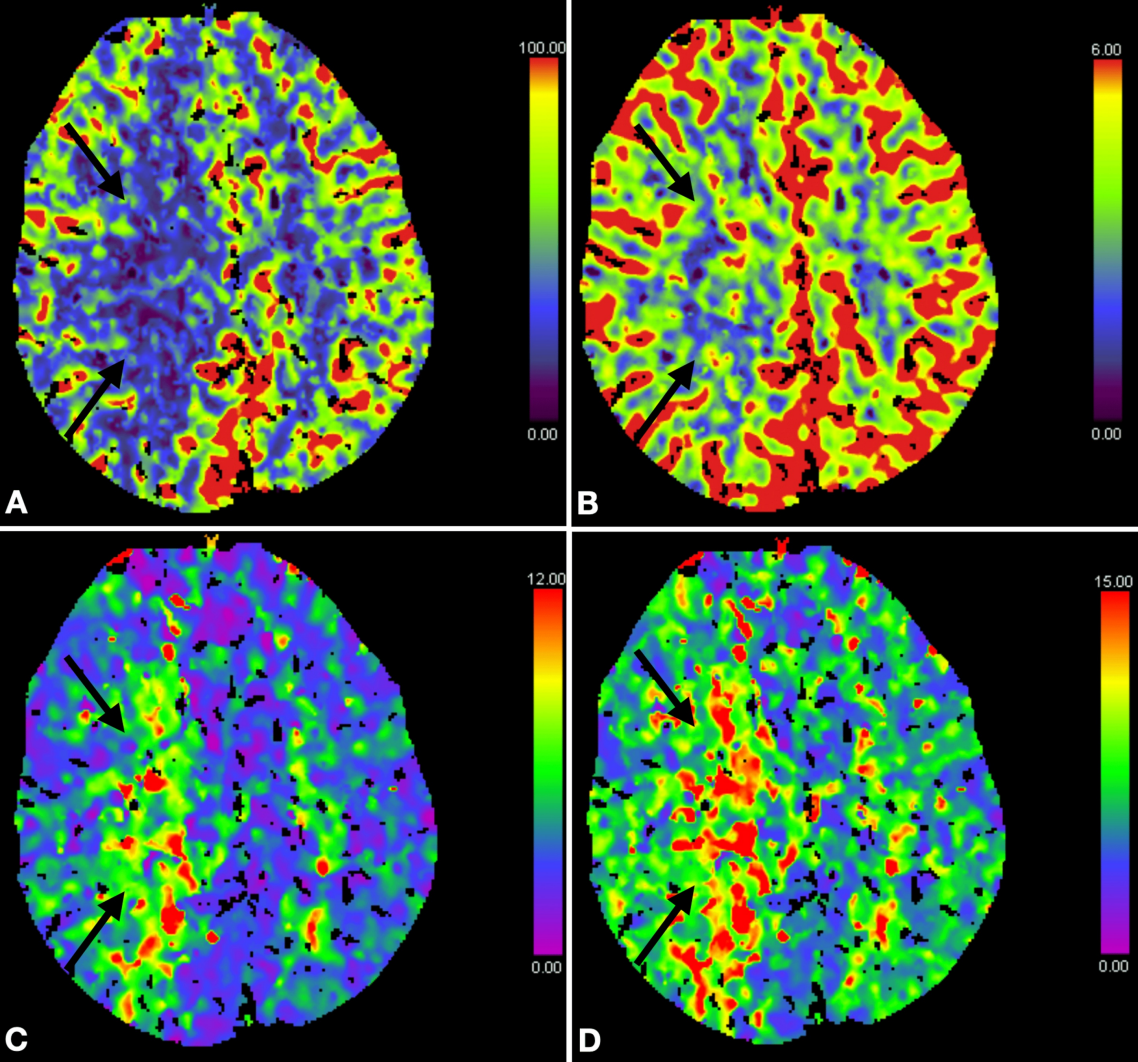
Computed tomography perfusion maps (A) Cerebral blood flow, (B) Cerebral blood volume, (C) Time to maximal perfusion, and (D) Time to peak perfusion. A perfusion deficit is evident in the posterior and internal watershed zones on the right (arrows).

CT perfusion (Figure 2) demonstrated a perfusion deficit in the posterior and internal watershed zones on the right, which was no longer compensated by cerebral blood flow (CBF)/cerebral blood volume (CBV) in the demarcated ischaemic region.

Globally delayed, but still volume-compensated, CBV was observed in the left hemisphere, as well as in the anterior watershed zones on the right.

**Figure 3:**
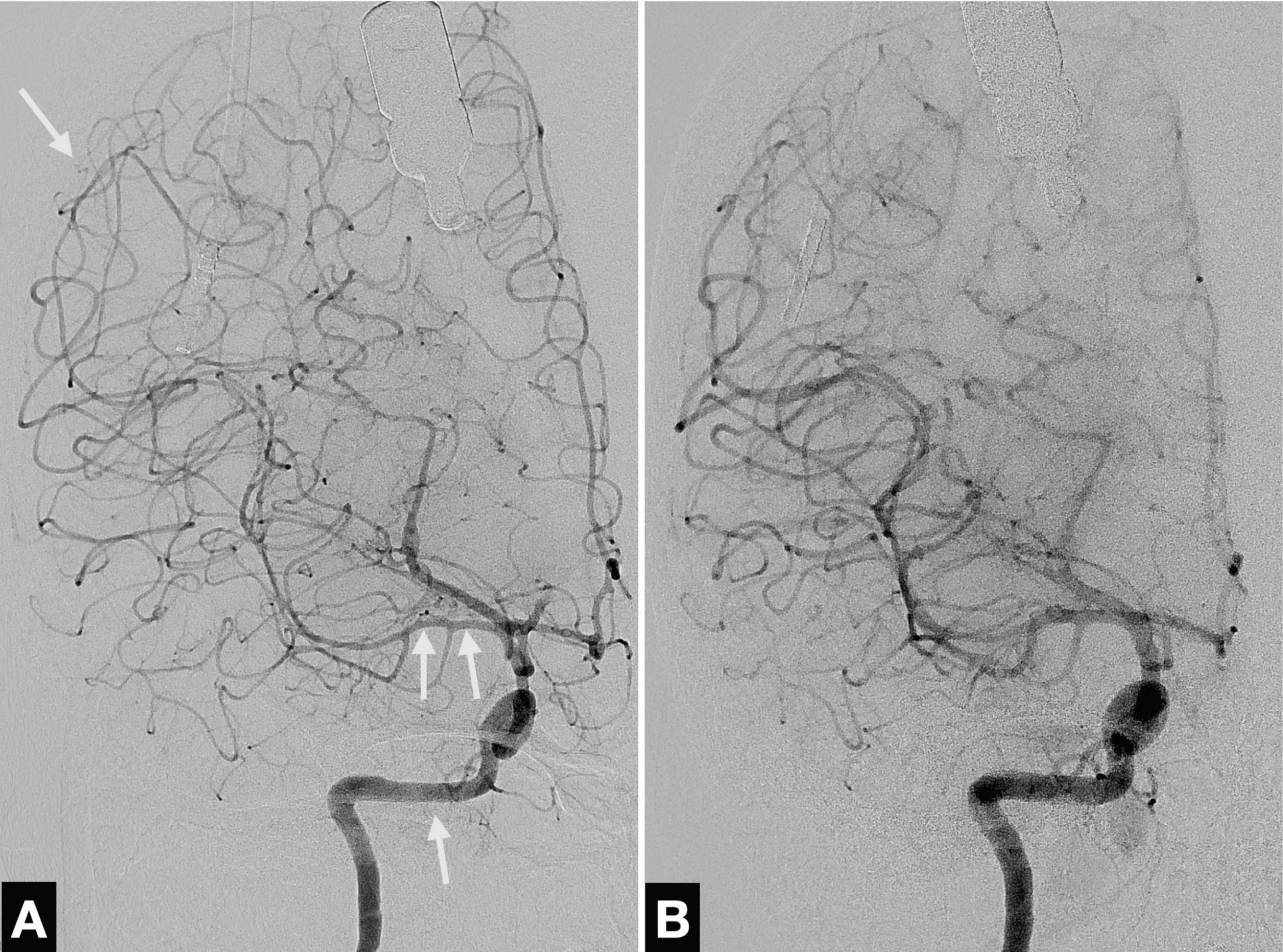
Digital subtraction angiography following initial imaging in the anteroposterior plane (A) Marked vasospasms (arrows) of the right internal carotid artery and intracranial vessels prior to spasmolysis. (B) Improved vessel calibres after intra-arterial spasmolysis with 6 mg nimodipine.

Due to the generalized vasospasm and signs of increased intracranial pressure, including cerebral oedema, constricted sulci, stretched optic nerve, and optic disc protrusion (papillary oedema), the patient was transferred to our angio-suite to undergo intra-arterial spasmolysis with 6 mg nimodipine. Spasmolysis significantly reduced the irregularities in the diameter of all the intracranial vessels (Figure 3).

Additionally, an external ventricular drain (EVD) was inserted to monitor the intracranial pressure and facilitate cerebrospinal fluid drainage. The opening pressure at the time of EVD insertion was 26 mmHg. For further monitoring, two probes were installed after spasmolysis: one to monitor CBF/perfusion, and a second to measure oxygen partial pressure and temperature in the tissue.

The treatment strategy focused on maintaining brain tissue oxygenation (ptiO₂) above 15 mmHg as the primary therapeutic goal. Blood pressure targets were adapted to support this ptiO₂ threshold. To optimise cerebral perfusion, norepinephrine was administered. In cases where ptiO₂ values fell below the threshold despite haemodynamic support, intra-arterial spasmolysis with nimodipine was performed.

**Figure 4:**
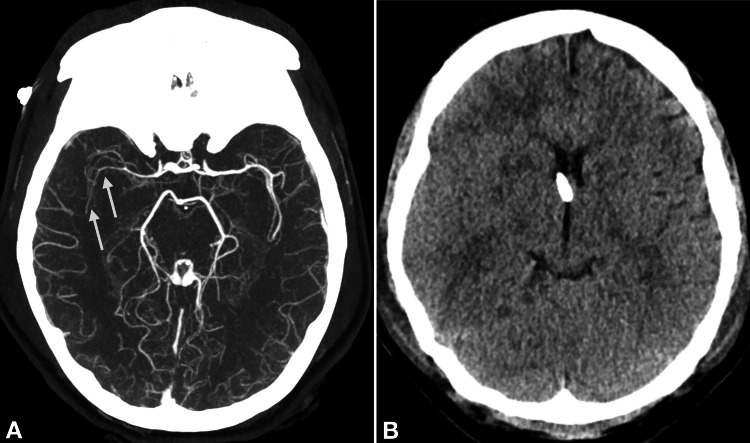
Second computed tomography scan performed 12 hours after initial imaging (A) Persistent vasospasm in the right middle cerebral artery on contrast computed tomography angiography (arrows). (B) Non-contrast CT shows progressive swelling of the right cerebral hemisphere; an external ventricular drain is visible.

Despite multimodal treatment, the patient's neurological condition continued to deteriorate. A second native brain CT with CT angiography and CT perfusion was acquired 12 hours following the initial scan. The native CT (Figure 4B) showed progressive brain swelling and clear delineation of infarcts in the right basal ganglia.

The CT angiography (Figure 4A) revealed a persistent severe intracranial vasospasm, especially of the MCA and its right-hand branches, associated with perfusion deficits in the border zone of the right hemisphere.

Intra-arterial spasmolysis with 6 mg nimodipine was therefore repeated, with moderate success.

**Figure 5:**
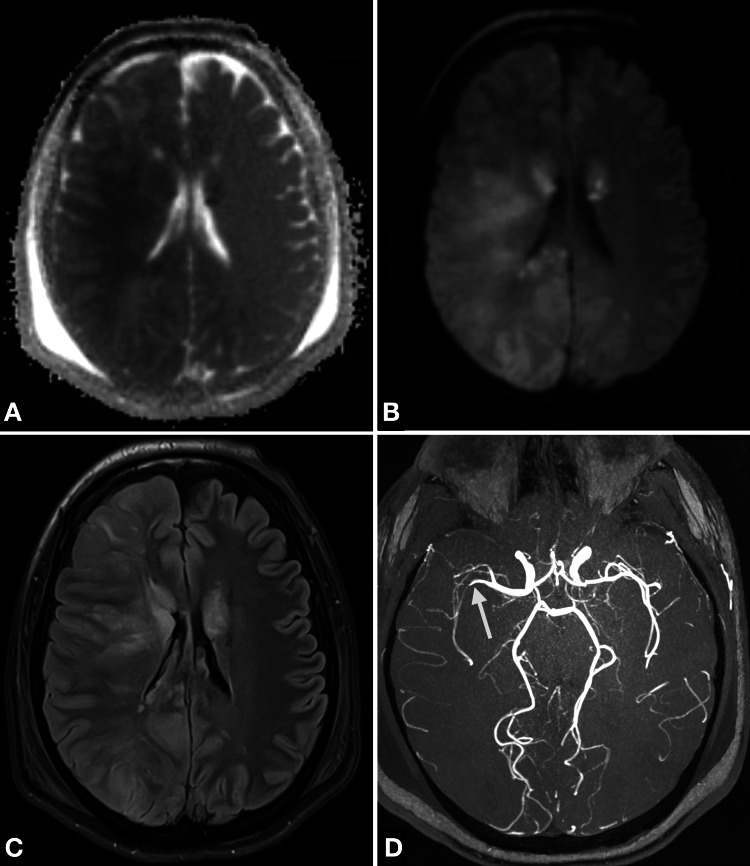
Brain magnetic resonance imaging for prognostic evaluation (A) Apparent diffusion coefficient and (B) Diffusion-weighted imaging (b1000) demonstrate bilateral supratentorial diffusion restriction, most pronounced in the right parieto-occipitotemporal region and basal ganglia. (C) Fluid-attenuated inversion recovery sequence demonstrating hyperintense signals corresponding to the areas of diffusion restriction. (D) Magnetic resonance angiography showing residual vasospasms with calibre irregularities in the right middle cerebral artery and both the anterior cerebral arteries (arrows).

Magnetic resonance imaging (MRI) of the brain and spine was performed on the third day of admission to assess the likely outcome. The imaging of the spine aimed to rule out any additional underlying causes of the severe vasospasm, such as spinal bleeding or complications related to the PDA. The MRI confirmed the progression of ischaemia (Figures 5A-5C), with involvement of supra- and infratentorial regions across all vascular territories and additionally identified a subarachnoid haemorrhage within the caudal dural sac of the spinal cord.

Furthermore, signs of secondary hypoxic encephalopathy were observed, presenting as a bilateral pattern with notable involvement of the thalami. Additional infarcts were detected, characterized as small-volume, sharply demarcated ischaemic lesions. Magnetic resonance angiography (Figure 5D) shows persistent vasospasms, with marked calibre irregularities of the right MCA and both anterior cerebral arteries.

Given the poor prognosis and continued progression of ischaemia due to the persistent RCVS, the focus of treatment was shifted towards palliative care. Despite the best efforts from physicians, the patient died six days after hospitalization. This case underscores the importance of early recognition and aggressive management of RCVS in the postpartum setting, particularly when complicated by haemorrhagic shock and massive transfusion. Clinicians should remain vigilant for neurological deterioration in postpartum patients, as vasoactive agents and transfusion-related factors may precipitate severe cerebral vasospasm. Early imaging, interdisciplinary coordination, and timely intervention such as intra-arterial spasmolysis, are critical to optimise outcomes. Awareness of RCVS as a potential complication of life-saving interventions in obstetric emergencies is essential for reducing morbidity and mortality.

## Discussion

This case highlights the potentially severe complications of RCVS, especially in postpartum women, and emphasizes the need for clinicians to be aware of the numerous triggers, such as massive transfusion and vasoactive drugs.

The underlying mechanisms contributing to RCVS have yet to be adequately elucidated and are subjects of ongoing research. However, it is well established that dysregulation of vasodilatory responses within the arterial cerebral endothelium plays a significant role in the pathophysiology [6]. In addition to internal factors like female sex and the puerperium [1,6-8,10], which are linked to adverse outcomes, external factors, including the use of RBC products [5,9,12,13] or vasoactive medications [3,4,7,9] also contribute significantly. 

Smith et al. [12] proposed several potential mechanisms for the harmful effects of blood transfusions. First, the transfused RBCs may be depleted of nitric oxide, an endogenous vasodilator, which can lead to increased vasospasm. Second, the presence of transfused RBCs may initiate an inflammatory response, potentially due to their pro-inflammatory characteristics, which could worsen the vasospasm. Third, the surplus iron found in the transfused RBCs may increase oxidative stress, while cytokines produced during storage could exacerbate ischaemia. In our patient, a total of seven units of RBCs were administered in less than 24 hours, qualifying as a massive transfusion.

Notably, more than half of RCVS cases occur during the puerperium or following exposure to vasoactive agents [9,14]. Our patient required vasoactive drugs, such as norepinephrine, to maintain adequate blood pressure. These drugs can induce a transient dysregulation of cerebral vascular tone, leading to segmental narrowing of the arteries [1,7,9,15]. Additionally, by triggering aberrant sympathetic overactivity, they may cause excessive sympathetic discharge, which subsequently disrupts the regulation of the cerebral vascular tone [1,7,9,15,16]. The exact mechanism by which these drugs cause vasospasms remains unclear, but they are recognized as significant precipitating factors in the development of RCVS.

RCVS predominantly affects women aged 20 to 50 years, and the postpartum period is recognized as a significant trigger [3,7,9,17]. Studies suggest that seven to nine percent of women develop RCVS during this period [17]. In a case series, most fatal cases occurred an average of 7.7±5 days after childbirth, which corresponds to the onset of symptoms in this case [6]. The role of hormonal imbalances, particularly related to pregnancy, is acknowledged. However, direct evidence linking hormonal imbalance to the disease's onset or progression remains lacking, highlighting the need for further research into these mechanisms.

The initial laboratory results showed a high anion gap lactic acidosis with a pH of 7.040, reflecting severe metabolic stress. While acidosis itself is not considered a direct cause of RCVS, such profound derangements indicate underlying shock and hypoxia, which are known to provoke sympathetic activation, endothelial dysfunction, and impaired cerebral autoregulation [4,15]. These mechanisms may act synergistically to promote cerebral vasospasm and trigger RCVS.

Our patient presented with several of these identified risk factors, which likely contributed to her rapid clinical deterioration. She was in the postpartum period, a recognized high-risk phase for the development of RCVS, and had undergone a massive transfusion. Additionally, vasoactive drugs such as norepinephrine were administered to maintain adequate blood pressure and cerebral perfusion, further compounding the risk of dysregulated cerebral vascular tone and vasospasm. The combination of these internal and external factors underscores the complex interplay of mechanisms that likely accelerated the progression of ischaemic damage and her subsequent decline.

The relationship between RCVS and vaginal bleeding requires further examination. While hypertension is common at the onset of RCVS [1,3,4,6,15], it can also cause bleeding from a vulnerable site such as a uterine suture, hence causing the haemorragic shock. However, it is equally plausible that the haemorrhagic shock itself triggered the development of RCVS. Significant blood loss may impair cerebral autoregulation through hypovolaemia and systemic hypoperfusion, initiating compensatory vasoconstriction [4,15]. This effect is further amplified by the administration of vasoactive agents and massive transfusion, both of which can promote endothelial dysfunction, inflammation, and oxidative stress. Moreover, postpartum uterine bleeding typically occurs around the second week after delivery [18-20], which is consistent with the timing in our patient. Additionally, the patient's clinical course prior to the haemorrhagic event, apart from a single severe headache episode, did not match with the classical presentation of RCVS, such as recurrent thunderclap headaches. In the end, the precise causal chain remains unclear.

Several strategies can be considered to improve the prevention and early recognition of transfusion-triggered RCVS and warrant further evaluation in clinical practice. Central to this is a more nuanced and individualized approach to transfusion management. Abrupt haemoglobin correction may act as a physiological stressor, precipitating vascular dysregulation [5]. Therefore, gradual correction with close monitoring of haemodynamic parameters should be explored as a potential risk-reducing strategy. Post-transfusion surveillance could also benefit from standardized neurological assessments, with a low threshold for early neuroimaging in the event of a headache or other neurological symptoms. Specifically, non-contrast CT can exclude acute haemorrhage, while CT angiography can detect vasoconstriction and early signs of vasogenic oedema. 

Our case report highlights the importance of early recognition and intervention, as timely management may significantly alter outcomes for patients with atypical or severe manifestations of RCVS. Previous reports note that most patients with RCVS have an excellent prognosis, with a low mortality rate (between 1% and 5%) [6]. In the case of fatal outcomes, initial imaging showed abnormal findings such as oedema, infarction, and intracranial or subarachnoid haemorrhage [6]. Similarly, in our patient, the initial CT imaging revealed ischaemia and cerebral oedema. Therefore, close monitoring in the intensive care unit is needed to improve outcomes in patients with these initial findings.

Incorporating patient-specific risk factors such as pre-existing vascular disease, hormonal influences, and other comorbidities into transfusion decisions may improve risk stratification and help detect RCVS early. Institutional protocols that define transfusion thresholds, establish blood pressure targets, and discourage the use of vasoactive agents when avoidable could further reduce the risk. As RCVS remains underrecognized, developing evidence-based guidelines and advancing research into its pathophysiology are essential to improve prevention and patient outcomes. Vasopressin, which raises blood pressure without adrenergic activation, may offer a safer alternative for managing RCVS and merits further study. There are still no accepted guidelines for the management of RCVS, due to the lack of randomized controlled trials and standardized treatment protocols, highlighting the need for future research in this area.

## Conclusions

Our case highlights the complex interplay between life-saving interventions, such as transfusions and vasoactive medications, and secondary complications like RCVS that can lead to catastrophic outcomes. In women, the postpartum period represents a particularly vulnerable time for cerebral autoregulation, and interventions such as massive transfusion and vasopressor use may inadvertently precipitate severe neurological complications like RCVS. Improving awareness, early detection, and interdisciplinary management of RCVS in postpartum settings is crucial to improve patient outcomes. Implementing structured neurological assessments and using early neuroimaging tools, especially CT with CT angiography and CT perfusion, can facilitate early diagnosis. Haemodynamic monitoring with attention to blood pressure targets and gradual haemoglobin correction may help minimise cerebral stress. However, as this report describes a single clinical case, generalisation is limited. Larger studies are needed to confirm these observations and support the development of standardised protocols. Future investigations should focus on delineating hormonal contributions to postpartum RCVS, evaluating transfusion thresholds and protocols, and exploring optimal pharmacologic strategies for vasospasm management. The development of evidence-based guidelines tailored to high-risk obstetric populations remains an urgent need.
